# A Herpes Simplex Virus Thymidine Kinase-Induced Mouse Model of Hepatocellular Carcinoma Associated with Up-Regulated Immune-Inflammatory-Related Signals

**DOI:** 10.3390/genes9080380

**Published:** 2018-07-27

**Authors:** Zhijuan Gong, Qingwen Ma, Xujun Wang, Qin Cai, Xiuli Gong, Georgi Z. Genchev, Hui Lu, Fanyi Zeng

**Affiliations:** 1 Shanghai Institute of Medical Genetics, Shanghai Children’s Hospital, Shanghai Jiao Tong University, Shanghai 200040, China; gongzj@hotmail.com (Z.G.); qwma1213@163.com (Q.M.); caiqin417@hotmail.com (Q.C.); gongxl@shchildren.com.cn (X.G.); 2 Department of Histo-Embryology, Genetics and Developmental Biology, Shanghai Jiao Tong University School of Medicine, Shanghai 200025, China; 3 Key Laboratory of Embryo Molecular Biology, Ministry of Health & Shanghai Key Laboratory of Embryo and Reproduction Engineering, Shanghai 200040, China; 4 SJTU-Yale Joint Center for Biostatistics, School of Life Science and Biotechnology, Shanghai Jiao Tong University, 800 Dongchuan Road, Shanghai 200240, China; jameswong.tju@gmail.com (X.W.); georgi.z.genchev@gmail.com (G.Z.G.)

**Keywords:** herpes simplex virus thymidine kinase, mouse model, hepatocellular carcinoma, transcriptome analysis

## Abstract

Inflammation and fibrosis in human liver are often precursors to hepatocellular carcinoma (HCC), yet none of them is easily modeled in animals. We previously generated transgenic mice with hepatocyte-specific expressed herpes simplex virus thymidine kinase (*HSV-tk*). These mice would develop hepatitis with the administration of ganciclovir (GCV). However, our HSV-tk transgenic mice developed hepatitis and HCC tumor as early as six months of age even without GCV administration. We analyzed the transcriptome of the *HSV-tk* HCC tumor and hepatitis tissue using microarray analysis to investigate the possible causes of HCC. Gene Ontology (GO) enrichment analysis showed that the up-regulated genes in the HCC tissue mainly include the immune-inflammatory and cell cycle genes. The down-regulated genes in HCC tumors are mainly concentrated in the regions related to lipid metabolism. Gene set enrichment analysis (GSEA) showed that immune-inflammatory-related signals in the HSV-tk mice are up-regulated compared to those in Notch mice. Our study suggests that the immune system and inflammation play an important role in HCC development in HSV-tk mice. Specifically, increased expression of immune-inflammatory-related genes is characteristic of HSV-tk mice and that inflammation-induced cell cycle activation maybe a precursory step to cancer. The HSV-tk mouse provides a suitable model for the study of the relationship between immune-inflammation and HCC, and their underlying mechanism for the development of therapeutic application in the future.

## 1. Introduction 

The liver plays a pivotal role in the metabolic homeostasis in the human body, and a wide variety of factors can affect the liver leading to steatohepatitis, fibrosis, cirrhosis, and cancer [1,2]. Hepatocellular carcinoma (HCC) is the second most common cause of cancer-related death in the world [3], whose pathogenesis is still not completely understood. For the study of the pathogenesis of HCC and to investigate the effects of potential therapies, a few mouse HCC models have been developed. Such genetically engineered mouse models have greatly facilitated studies of gene function in HCC development and are also valuable tools for understanding the underlying biological mechanisms of tumorigenesis.

Herpes simplex virus thymidine kinase (*HSV-tk*) expression in specific tissue cells has been used for cell-specific ablation [4,5,6,7,8]. Unlike mammalian nucleoside kinase, *HSV-tk* efficiently phosphorylates a range of nucleosides and analogs such as ganciclovir (GCV). GCV is not toxic in its unmodified form but causes cell death when phosphorylated intracellularly [9,10]. In our previous work [11], we generated transgenic mice with hepatocyte-specific expressed *HSV-tk* driven by a serum albumin promoter/enhancer to serve as a mouse model for liver damage research. 

In this study, we established three more lines of HSV-tk mice for further exploration of liver damage in this mouse model. Interestingly, all three lines of transgenic HSV-tk mice developed liver cancer even without GCV administration, which could indicate that *HSV-tk* as a kinase gene might contribute to tumorigenesis. The mechanisms linking *HSV-tk* induced oncogenesis, however, are mostly unknown. It is unclear whether HCC in HSV-tk mice is induced by cell transformation directly and evokes a specific carcinogenic phenotype, or through prolonged steatohepatitis. Here, we examined the transgene integration sites of these mice and compared the pathologic changes in livers from wild-type (WT) and HSV-tk transgenic mice for more than one year.

Computational methods have found increased application in analyzing the complexities underlying experimental studies and have been used in diverse areas of biomedical and cancer research such as to investigate expression profiles [12,13] and genomic alterations [14,15]; the role in miRNA in tumor origin localization [16,17]; in human HCC [18,19]; in the prediction of disease and cancer genes [20,21]; and in cancer prognosis [22,23]. To study the possible causes of HCC, we investigated the transcriptome of HSV-tk mouse liver tissues with HCC and hepatitis using computational analysis of microarray data. We report here our data that may elucidate the possible roles of immune-inflammatory and cell cycle genes in the occurrence and development of *HSV-tk*-related HCC. Our result suggests that such HSV-tk mouse as a novel animal model could provide a useful and clinically-relevant platform for further HCC clinical study.

## 2. Methods

### 2.1. Generation of HSV-tk Transgenic Mice

Generation of a hepatocyte-specific expressed HSV-tk transgenic mouse was achieved as described earlier using pronuclear microinjection [11]. Briefly, Kunming (KM) mice were used in this study to generate three HSV-tk transgenic mouse lines. A 4.2-kb pLLTK fragment containing murine serum albumin (ALB) gene promoter/enhancer was used to construct an *HSV-tk* expression unit which was microinjected and manipulated to generate transgenic mice. The integration of *HSV-tk* was confirmed primarily by PCR, and the integration sites of *HSV-tk* were investigated by inverse PCR. All mice were kept in rooms at a constant temperature and humidity in a 12 h light/dark cycle. The experimental animal permit number is “SYXK (Shanghai) 2013-0051”. This study was approved by the Animal Experimentation Committee of the Shanghai Children’s Hospital for Experimental Animals.

Inverse PCR was performed as described previously [24] with modification. Five µg of genomic DNA of HSV-tk mouse was digested with *Dra*I and *Eco*RI restriction endonuclease enzyme respectively and circularized by T4 DNA ligase. The circularized DNA was further used as a template for nested-PCR. The first-round PCR products were used as a template for second-round PCR; primer sequences are shown in Table 1. The PCR products were purified and sent for sequencing (BGI, Shenzhen, China). Sequencing results were aligned with pLLTK plasmid sequences and mouse genome respectively in National Center for Biotechnology information (NCBI, https://www.ncbi.nlm.nih.gov/) using Blast search (https://blast.ncbi.nlm.nih.gov/Blast.cgi).

### 2.2. Immunofluorescence Analysis

The frozen sections (10 μm thickness each) from mice liver were used for immunofluorescence studies. The sections were incubated with rabbit-anti-mouse primary monoclonal antibodies for either Ki67 (Abcam, Cambridge, UK) or CK-19 (Abcam), respectively, both in 1:100 dilutions, at 4 °C overnight; after washing 3 times, the sections were stained with Alexa 488 fluorescence labeled goat-anti-rabbit second antibody (Abcam) at 1:300 dilutions for 30 min, room temperature (RT). After brief washing, the sections were observed under a fluorescence microscope Leica, DFC310 FX (Wetzlar, Germany).

### 2.3. The Incidence of HCC and Histopathological or Immunohistochemistry Analysis in HSV-tk Mice

Three or more HSV-tk transgenic mice and WT mice were sacrificed starting from the age of 3-month-old, and then every three months until the mice reached an age of over 13 months. Before sacrifice, blood was collected from the orbital plexus for serologic tests of the values of ALT (alanine aminotransferase) and AST (aspartate aminotransferase) using clinical chemistry Analyzer (Abbott Architect c8000, Chicago, IL, USA). The livers were examined for gross lesions, then fixed with 10% buffered formaldehyde (formaldehyde 4%, NaH_2_PO_4_·H_2_O 0.4%, Na_2_HPO_4_ 1.3%), the liver tissue from each lobe and tumors were embedded in paraffin, sectioned, and stained with hematoxylin and eosin (H&E). Immunohistochemistry was performed following the instruction of the procedure for each antibody. Sections prepared from the paraffin-embedded blocks were stained with a CD3 antibody (Beyotime, Shanghai, China) and CD11b (Abcam), both in 1:200. For microarray analysis and immunofluorescence, non-tumorous tissues and individual tumors were snap frozen in liquid nitrogen and stored at −80 °C until used. Histopathological changes in these livers were observed by two pathologists independently.

### 2.4. Microarray Data Analysis

Microarray analysis was performed in tumor and hepatitis tissue and in liver tissue from an age-matched control group (KM mice without HSV-tk). The gender of the mice, serologic tests, and overall health condition are included in Supplementary Table S1. RNA was isolated from frozen tissue using the miRNeasy Mini Kit (Qiagen, Duesseldorf, Germany) and all samples for molecular analysis were paired with histopathology samples for histopathological and immunohistochemistry analysis. RNA was hybridized on Gene Chip Mouse Gene 1.0 ST Array (Affymetrix, Santa Clara, CA, USA) and scanned on an Affymetrix Scanner 3000; the data were obtained using the Gene Chip Command Console Software (AGCC, version 1.1). 

Gene expression data were normalized across all 12 samples (4 samples each group) using the robust multiarray analysis (RMA) methodology by the R *affy* package and the *Bioconductor* software (version3.7). Custom Chip Description File (CDF, mogene10stmmrefseqcdf_21.0.0) was downloaded (http://brainarray.mbni.med.umich.edu/Brainarray/Database/CustomCDF/CDF_download.asp) and installed to annotate the probe.

An unsupervised hierarchical clustering analysis was performed to evaluate the distance between samples. There were marked differences in global gene expression which segregated tumor from both steatotic hepatitis and WT liver. The *limma* package was used to call the differential gene expression. T-statistics derived from *limma* were used to represent the significance of differential expression and directions.

### 2.5. Gene Functional Enrichment Analyses

We performed principal component analysis (PCA) on the normalized data and clustered the samples based on gene expression. PCA was used as linear transformation to reduce the dimension of the data to find orthogonal variables (principal components, PC) that describe the variability in the data. For the exploration of gene functions related to PC1, the top 3000 genes correlated with PC1 were analyzed. Gene Ontology (GO) and Kyoto Encyclopedia of Genes and Genomes (KEGG) datasets were used to annotate the genes. The heatmap was generated to illustrate the enrichment significance of the gene functions.

### 2.6. Comparison of HCC Gene Expression Profiles between HSV-tk and Notch Transgenic Mice

To compare HCC gene expression profiles of *HSV-tk* and *Notch* mouse models, we performed analysis of the microarray data by Gene Set Enrichment Analysis (GSEA) [25] on a Notch mice microarray dataset GSE33486 [26] downloaded from the GEO database at http://www.ncbi.nlm.nih.gov/gds/. We identified genes that were differentially expressed between the two models by (1) using the *limma* package-calculated tumor versus normal expression gene *t*-test in the two data, named T-geo and T-our respectively; (2) subtracted T-geo to T-our; (3) compared and contrasted the up-regulated and down-regulated genes and signaling pathways by GSEA.

## 3. Results

### 3.1. Generation and Characterization of HSV-tk Mice

**Figure 1:**
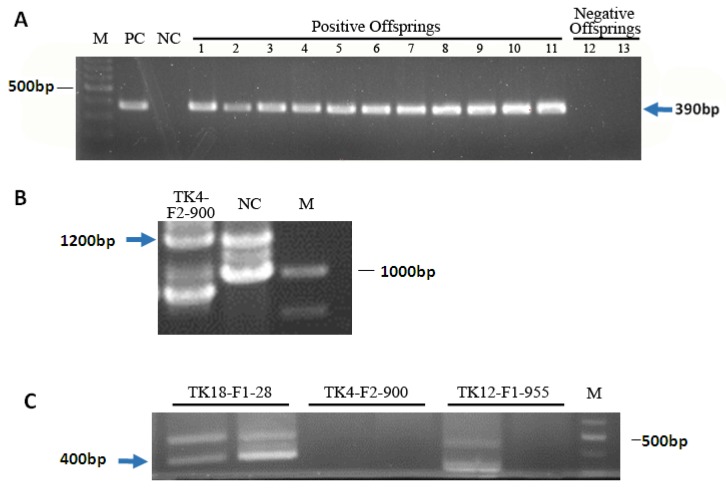
Analysis of integration status in mouse and the integration sites of *HSV-tk*. (**A**) PCR analysis of the Herpes simplex virus thymidine kinase (*HSV-tk*) gene in offspring of founder mice. PC, positive control (pCMV-TK vector), size at 390 bp; NC, negative control (wild-type KM mice); 1–11, 11 offspring with positive HSV-tk integration; 12–13, 2 offspring without HSV-tk integration; M, 100 bp marker. (**B**,**C**) iPCR analysis of HSV-tk transgenic mice flanking sequence of integration sites. (**B**) The PCR product of ligated *Dra*I digestion of genomic DNA. NC, negative control; M, 1 kb marker. (**C**) The PCR product of ligated *Eco*RI digestion of genomic DNA. M, 100 bp marker.

HSV-tk transgene founder mice with the integration of *HSV-tk* were proved by PCR analysis (data not shown). Transgenic lines were established from these female founders by mating them with wild-type male mice. We created three lines of transgenic mice with hepatocyte-specific expression of *HSV-tk* by using the mouse albumin promoter/enhancer element to drive HSV-tk expression. All three lines were established from F_0_ females. PCR analysis of the *HSV-tk* gene in offspring of founder mice was performed and confirmed that that transgene was transmitted to the offspring (Figure 1A). The inverse PCR procedure permits the rapid amplification of unknown sequence flanking adjacent to *HSV-tk* integration sites. The nested-iPCR was performed, and we obtained 1200 bp and 400 bp specific DNA fragment (Figure 1B,C). The DNA fragment was purified and sequenced. By NCBI sequence alignment, the integration sites of the three lines were determined (Table 2). *HSV-tk* in TK4-F_2_-900 was found to be integrated into C region of chromosome 8, located between Cerebellin-1 precursor gene and uncharacterized gene C16 or F18 homolog. Transgenic integration of TK5-F_1_-28 was in E region of chromosome 5, located in the intron of *Rufy3* which is known to suppress formation of surplus axons for neuronal polarity [27], and is indicated in relating to carcinoma [28,29]. The integration site of *HSV-tk* in TK11 line (TK11-F_4_-104) is located on chromosome 11 in the intron of *abca13* which encodes a transporter protein [30].

### 3.2. The Incidence of HCC and Histopathological Analyses in HSV-tk Mice

**Figure 2:**
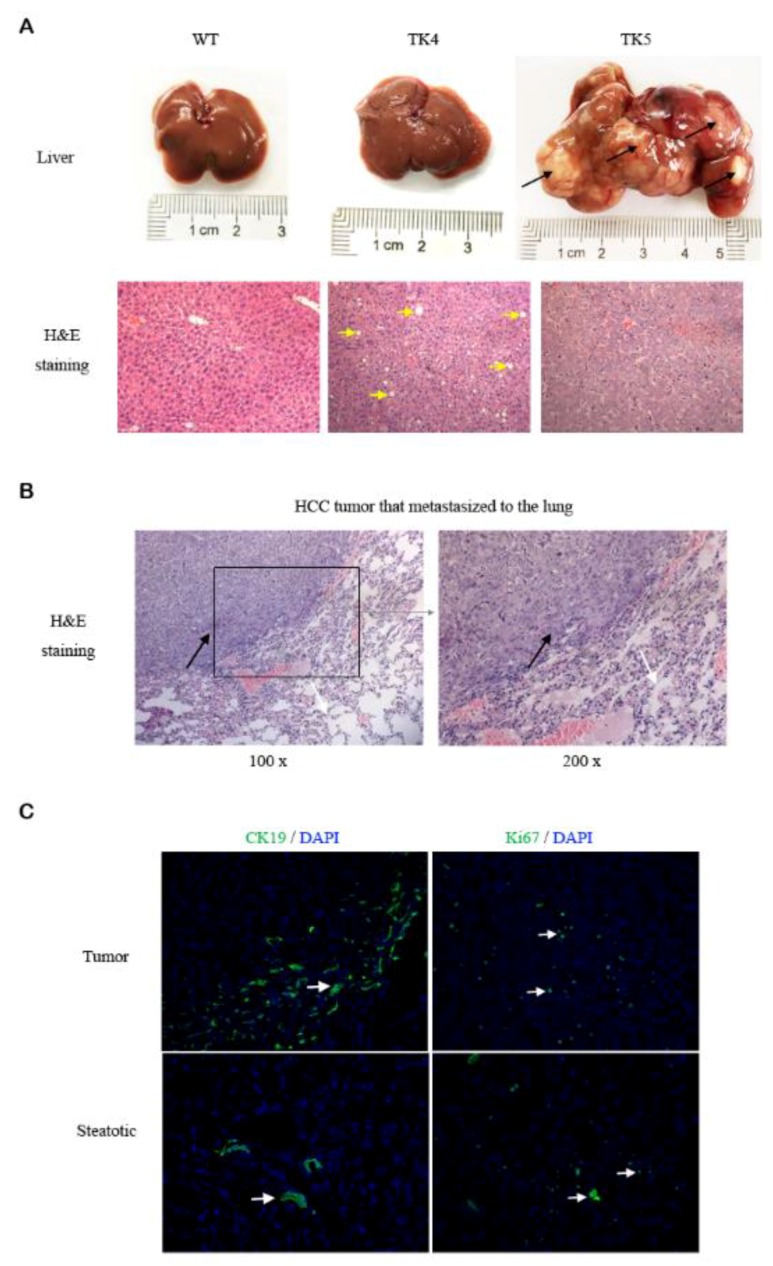
Histopathological analysis of hepatic steatosis and HCC in HSV-tk mice at 12 months of age. (**A**) Upper row images: gross tissue image of liver specimens from wild type (WT), TK4 and TK5 mice. No apparent change was observed in the liver of WT; steatosis was seen in the liver of line TK4 mouse and multiple tumors developed (black arrows) in the liver of TK5 mouse. Lower row images: histological analysis of the relevant WT, lines TK4 and TK5 mice liver. Note the marked steatotic changes in the TK4 liver (yellow arrows), and poorly differentiated tumor cells in the TK5 liver (H&E staining, Magnification, ×200); (**B**) HCC transferred to the lung in HSV-tk mice. Histological analysis of HCC transferred to the lung of a TK5 mouse at 13 months of age (HCC: black arrows, lung tissue: white arrows) (H&E staining); (**C**) Immunofluorescent observation for CK19 and Ki67 in HCC and steatotic tissue. In the tumor, Ki67 staining confirmed a high proliferative index, while only a small portion of cells was positive for CK19. In steatotic tissue, positive CK19 cells were limited in biliary ducts, and there were a few proliferation hepatocytes and inflammation cells. (Magnification, ×200).

Tumorigenesis was explored by serum ALT and AST test prior to sacrifices and necropsy for gross tumor nodes and histopathological analysis. The tumors were noted to be accompanied by increased serum ALT and AST. In all of the three lines of HSV-tk mice examined, no tumor was detected in livers during the first three months. The number and size of visible hepatic tumors in HSV-tk mice increased with aging and varied among the three lines. For example, tumors could be detected as early as six months in all mice from the TK5 line, but the onset of first detectable tumor in TK4 and TK11 lines was at nine months. On the other hand, at 12 months of age, all mice in lines TK5 and TK11 had an advanced tumor with a diameter of at least 1 cm characterized by the disrupted lobular structure; however, only forty percent of line TK4 mice present liver tumors (Table 3). HCC was found in formalin-fixed tumor samples from all three lines of HSV-tk mice, and with a various histological pattern, from well to poorly differentiated. A significant proportion of steatotic hepatitis was found inside of the tumor tissue. Degenerated hepatocytes were detected in line TK4 mice with steatotic hepatitis at 12 months of age (Figure 2A). Two cases of HCC tumor with lung metastases (a primary feature of aggressiveness in a tumor) were found in line TK5 and TK11 mice at 13 months age (Figure 2B).

Ki67 immunofluorescent staining confirmed a high proliferative index in the tumors. A small portion of hepatic tumors cells was positive for cytokeratin 19 (CK19), a marker of bilinear lineage cells [31] (Figure 2C). To study whether immune cell infiltrated into the harvested tumor tissues for the transcriptome study, immunohistochemistry of different types of immune cell including T cells (CD3^+^) and granulocytes (CD11b^+^) were performed. Interestingly, CD3^+^ T cell and CD11b^+^ granulocytes were found to be present only in the hepatitis group, but not in the tumor and control group (Supplementary Figure S1), thus, this excluded the possibility that the up-regulation of immune/inflammatory-related genes in the tumors were caused by the immune cell infiltration into the harvested tumor tissues. 

**Figure 3:**
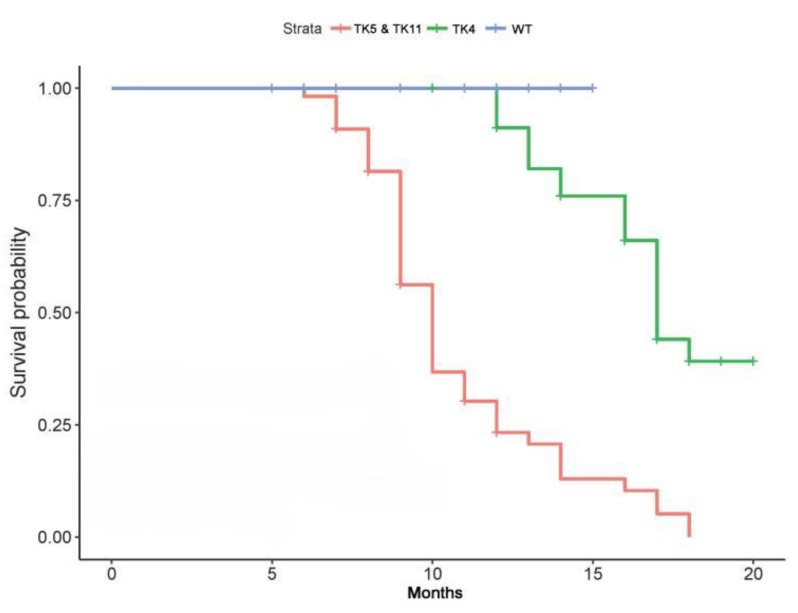
Analysis of onset of HCC development in three lines of HSV-tk transgenic mice. Kaplan-Meier plots showing the survival probability over time (months) of the three lines of HSV-tk mice. Thus, the survival signature was validated for discriminating between lines TK4, TK5, and TK11, as it was showing significant differences in survival time.

Using time and survival probability analysis of hepatic tumor development in HSV-tk mice, we found that whether *HSV-tk* was located in the intergenic region (TK4) or within the introns region of the gene (TK5 and TK11), the three lines of HSV-tk mice showed the presence of tumorigenesis. If the survival probability of WT mice is set to 1, survival probability of TK5 and TK11 mice has reached almost zero in 15 months of age, while the survival probability for TK4 mice is 0.3 in 20 months. The time of tumor development is at 6 months of age in TK5 and TK11 mice, which is significantly earlier than that in TK4 mice (Figure 3).

### 3.3. Immune/Inflammatory and Cell Cycle Abnormalities in HSV-tk Mice Tumors

**Figure 4:**
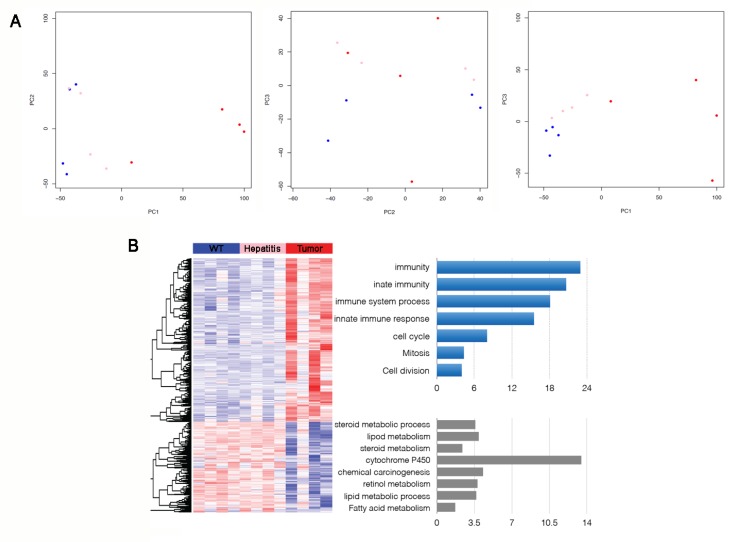
Immune-inflammatory and cell cycle abnormalities in HSV-tk mice tumors. (**A**) Principal components analysis comparing gene expression profiles of HCC, steatotic and WT liver. PC1 is the biggest principal component from the variance. The relationship between the principal component 1 (PC1), PC2, and PC3 (tumor: red dot, Steatotic: pink dot, WT: blue dot); (**B**) Functional analysis of differentially expressed genes of HCC, steatotic and WT liver. Heat map demonstrating down-regulated genes. Immune-inflammatory response and cell cycle-related genes were up-regulated in WT, steatotic and tumor samples. On the contrary, lipid metabolic genes were down-regulated. Red and blue colors indicate high and low gene expression, respectively; the top rank ordered processes are based on statistical significance.

Transcriptome analysis was performed using samples from *HSV-tk* tumors, steatotic hepatitis, and WT. Principal component analysis of the normalized data from all the comparisons illustrated the clear separation of the tumor and normal liver samples. The first three principal components (PC) that captures the majority of the variation in the data was used to visualize the spatial relationship of the WT, steatotic hepatitis, and tumor samples. Principal component 1 (PC1), with about 43% of the variation of the data captured, separates the three types of samples. PC2 with 13% and PC3 with 9% of the data captured, respectively. There was a significant difference in the expression profile of WT, steatotic hepatitis, and tumor, and the difference could be distinguished. Tumor samples were far separated from steatotic hepatitis and WT samples, and the three samples were in a progressive relation, and steatotic hepatitis and WT samples were more similar (Figure 4A).

The functional study of differentially expressed genes in tumor mice revealed the most significantly enriched fractions among genes up-regulated in hepatic tumors compared with steatotic hepatitis, and WT liver tissues were genes related to such GO biological processes as “immunity”, “innate immunity and immune system process”, and “cell cycle, mitosis” (Figure 4B). Numerous genes encoding immune-inflammatory response were induced in tumor samples. Some of the genes involved in innate immunity were up-regulated in the tumor samples, including *Tlr3*, *Clec7a*, *Isg20*, *Csf1*, *Irf7*, and *Lgals3*. Genes involved in immune response including the Major Histocompatibility Complex (MHC) class II antigen presentation as exemplified by *H2-Aa*, *H2-Ab1*, and *H2-Eb1* genes were up-regulated. *Cd74* gene involved in the formation and transport of MHC class II protein was also up-regulated. The *Alcam* gene, expressing on activated T cells was induced. Furthermore, genes encoding inflammatory response related proteins were increased, such as inflammatory caspase 4 (encoded by the *Casp4* gene), Nuclear factor-κB (encoded by the *Nfkb2* gene), whose inappropriate activation of NF-κB has been linked to inflammatory events. 

Remarkably, the majority of the discovered cell cycle-related genes that were up-regulated in tumorous samples compared with WT controls were involved in the advanced stages of mitosis. Immunofluorescent analysis was used to confirm the up-regulation of some of these genes involved in DNA replication (e.g., *Mcm6*) as well as the induction of *Ki67* (Figure 2C). Notably, antigen Ki67 is a nuclear protein that is associated with and may be necessary for cellular proliferation. The expression of key cell division regulators involved in mitotic spindle organization and chromosome segregation (e.g., *Mad2l1*) was simultaneously altered. Cell cycle-related genes (e.g., *E2f1*, *Src*), and master regulators of cell cycle checkpoint signaling pathways that required for DNA damage response and genome stability were also induced (e.g., *Brca1*, *Trp53*). It is well known that cell cycle-related genes are associated with tumor cell regulations [32]. We further explored these cell cycle-related genes in the Cancer Genome Atlas (TCGA) Liver Hepatocellular Carcinoma (LIHC)/HCC patient datasets. it is not surprising that most of these genes are also up-regulated in cancer compared to the normal (Supplementary Figure S2). At this time, there does not seem to be a significant difference in these normal HCC cases as compared to our model.

The set of genes that were down-regulated in tumor versus WT samples, and also in the tumor versus steatotic hepatitis samples, was enriched with lipid metabolic genes; the genes involved in fatty acid b-oxidation (e.g., *Acaa1a*, *Acadvl*, *Acox1*) were repressed (Figure 4B).

### 3.4. Comparison of HCC Gene Expression Profiles between HSV-tk and Notch Transgenic Mice

**Figure 5:**
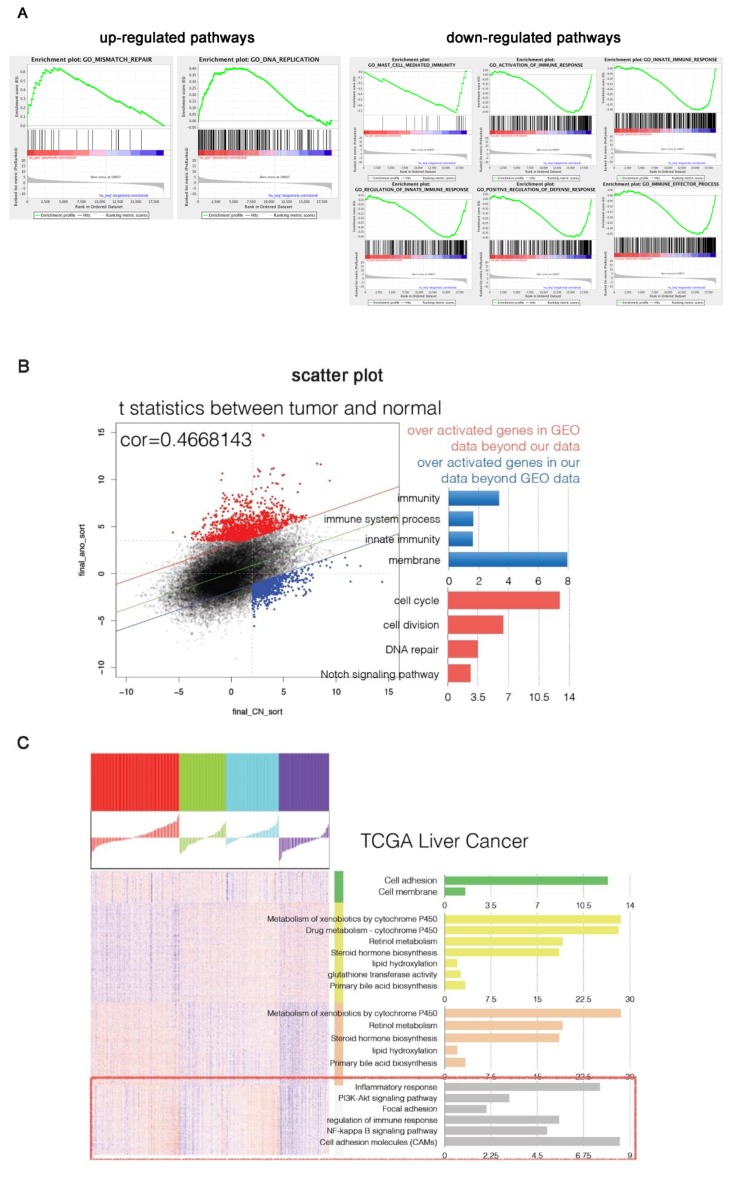
Comparison of HCC gene expression profiles between HSV-tk and Notch transgenic mice and the Cancer Genome Atlas (TCGA) data. (**A**,**B**) Gene set enrichment analysis (GSEA) comparing the up-regulated and down-regulated pathways of Notch and HSV-tk transgenic mice HCC. (**A**) Up-regulated and down-regulated pathways in Notch mice compared with HSV-tk mice. (**B**) HSV-tk mice immune-inflammation related signals were up-regulated compared to Notch mice. Increased expression of signals in Notch mice included cell cycle and DNA repair pathways. Over-activated genes in Notch mice beyond HSV-tk mice (red colors); Over-activated genes in HSV-tk mice beyond Notch mice (blue colors), respectively. (**C**) The change of pathways of human HCC in TCGA data. Immune-inflammatory reactions (red rectangle) in the human liver have high heterogeneity, are only highly expressed in some samples.

Tumor versus WT differentially expressed genes were compared between HSV-tk mice and published data for Notch transgenic mice. Gene set enrichment analysis was used to examine the up-regulated and down-regulated genes and signaling pathways. GSEA results showed that in the HSV-tk mouse immune-inflammation-related signals are up-regulated compared to those in Notch mice. Increased expression of signals in *Notch* mice included cell cycle and DNA repair pathways (Figure 5A,B). This suggests that increased expression of immune-inflammatory genes is one of the characteristics of HSV-tk mice. By analysis of the TCGA database of human liver cancer, we found that the signaling pathways of the immune-inflammatory reaction in human liver to have high heterogeneity, and only have high expression in some samples (Figure 5C).

## 4. Discussion 

In this study, we explored the possible mechanism of HCC development in HSV-tk transgenic mice. We generated three lines of HSV-tk mice and characterized their transgene integration and incidence of HCC, and performed histopathological analysis. Through microarray analysis, we explored the possible cell function and/or processes that are up- or down-regulated in the tumor mice. Also, we compare the global gene expression profile with other models such as Notch transgenic mouse.

We noted that the HSV-tk mice were thought to develop hepatitis with the administration of ganciclovir (GCV). However, our HSV-tk transgenic mice developed hepatitis and HCC tumor even without GCV administration. The number and size of visible hepatic tumors in HSV-tk mice increased with aging and varied among the three lines (Table 3). Although the exact mechanism is not known, we could hypothesize that they are due to the different integration site of the *HSV-tk* genes in these three lines. As described in Table 2, two of the three lines were integrated into introns of the genes, and the other in the intergenic region. The time point of first noticeable hepatic oncogenesis in line TK5 mice leads to an earlier high incidence of HCC (100%) at the age of six months. This time point is earlier compared to the time point of oncogenesis in transgene mice that have co-expression of c-Myc and TGF-α (8 months) and in mice expressing either ALB/c-Myc or the MT/TGF-α transgene alone (12 months) [33]. Histological analysis demonstrated that a significant proportion of hepatitis tissue had steatosis, and to a lesser extent—the tumor tissue. 

A lack of evidence implicating insertional mutagenesis for integration into promoter regions of genes involved in growth control and cancer implies that *HSV-tk* plays a positive role in hepatic oncogenesis. In our study, the expression of *HSV-tk* was detected in the liver and ectopically in the testis, but was not detectable in other tissues, such as kidney, pancreas, intestines, stomach, brain, skin and heart (by RT-PCR analysis and histopathological analysis, data not showed). It has been reported that the natural *HSV-tk* gene contains a cryptic internal testis-specific promoter, which results in testis expression of *HSV-tk* regardless of the promoter [34]. Histopathological analysis has revealed that testicular development was immature and almost no sperm was produced in these mice without GCV administration.

*HSV-tk* was integrated into the intron region of *Rufy3* in line TK5. In one recent paper when Wang et al. demonstrated P21-activated kinase-1 (*Pak1*) interacts with *Rufy3*, promotes *Rufy3* expression and *Rufy3*-induced gastric cancer cell migration [29]. To exclude the possibility of oncogenesis related to *Rufy3*, we explored the expression patterns of *Rufy3* and its interactive partner *Pak1*. *Rufy3* was not up-regulated in tumor compared to normal samples. Although *Pak1* was up-regulated in tumor compared to normal samples, it is also observed in TCGA liver cancer datasets, and thus not in a *Rufy3*-specific manner. Besides, we did not find any gastric cancer in these mice, and it is hard to conclude the involvement of *Rufy3*-related oncogenesis in TK5 mice line (Supplementary Figure S3). Please note that the two lines with intronic integration (TK5 and TK11) do have relatively higher HCC incidence, such that there might be an unknown interaction between the protein encoded by the disrupted gene and *HSV-tk* in these mice.

To better understand the underlying molecular changes that characterize the relationship between WT control liver and hepatic tumor in HSV-tk mice model, gene expression profiling in HCC and control samples of 12 months aged HSV-tk mice was analyzed. The results revealed that in this *HSV-tk* model, the genes up-regulated in tumors were enriched with the immune-inflammatory response and cell cycle-related genes. Whereas genes down-regulated in tumors were enriched with lipid metabolic genes, especially genes involved in fatty acid b-oxidation.

Numerous genes encoding immune-inflammatory response were induced in hepatic tumor samples; the most prominent among the up-regulated set—*Casp4*, *Nfkb2* which encodes proteins related to inflammatory response. Inappropriate activation of *nuclear factor κB* (*NF-κB*) has been linked to inflammatory events. Other genes involved in innate immunity such as *Tlr3*, *Clec7a*, *Isg20*, *Csf1*, *Irf7*, and *Lgals3*; or participated in MHC class II antigen presentation such as *H2-Aa*, *H2-Ab1*, and *H2-Eb1* were up-regulated. The *Alcam* gene expressing in activated T cells and the *Cd74* gene that is involved in the formation and transport of MHC class II protein were up-regulated too.

Chronic inflammation is an essential underlying condition for tumor development, accounting for approximately 20% of human cancer [35]. Activation of the hallmark of inflammatory responses factor—*NF-κB* [36], is frequently detected in tumors [37,38]. *NF-κB* is necessary to protect mature hepatocytes against immune attack [39] and genotoxic stress [40] and has a significant anti-apoptotic effect [41]. Pikarsky et al. [42] have shown that in the Mdr2-knockout mouse strain which spontaneously develops cholestatic hepatitis and HCC, up-regulation of *tumor necrosis factor-α* (*TNF-α*) induced *NF-κB* is critical for tumor promotion in later stages. Ablation of *NF-κB* activity in the hepatocytes led to a dramatic decrease in later stages of tumor progression [43]. The *Casp4* gene encodes a protein involved in immunity and inflammation [44]. Up-regulation of *Casp4* expression levels was associated with signaling pathways involved in apoptosis, inflammatory responses, and immune responses. Dysfunction of numerous immune-inflammation related genes suggests that the immune system has an important contribution to hepatic tumor occurrence and development in HSV-tk mice.

By analysis of the TCGA database of human HCC, we found that the signaling pathways of immune-inflammatory reaction in human liver show high heterogeneity. It would be worthwhile to conduct further study on whether HSV-tk mouse HCC model is similar to human HCC with high expression of immune-inflammatory signaling pathways.

Several the cell cycle and mitosis-related genes were up-regulated in HCC of HSV-tk mice, including DNA replication—*Mcm6*, high cellular proliferation index—*Ki67*, which was confirmed by immunofluorescent analysis (Figure 2C). Mitotic spindle organization and chromosome segregation—*Mad2l1* was simultaneously altered. Furthermore, the genes were also induced including master regulators of cell cycle checkpoint signaling pathways required for DNA damage response and genome stability—*Brca1*, *Trp53*, as well as cell cycle-related genes—*E2f1*, *Src*, *Tgfb1* and *Tgfb3*. 

Conner et al. [45] found that co-expression of *E2f1/c-Myc* further accelerates liver cancer development. When over-expressed, transcription factors *E2f1* and *c-Myc* are capable of driving quiescent cells into S phase in the absence of other mitogenic stimuli [46], in addition to providing a continuous proliferative signal, *E2f1* and *c-Myc* are also potent inducers of apoptosis and operate at least through one common pathway involving p53 [47,48]. Also, deregulated expression of *c-Myc* and *E2f1* is frequently found in cancer cells [49,50]. *E2f1*-mediated cell proliferation favored the predominance of diploid cells characteristic of the pre-neoplastic type of liver growth. Transduction of fetal mice with a feline lentiviral vector induces hepatic tumors which exhibit an E2F activation signature. Tumors exhibited highly significant up-regulation of E2F target genes, of which a majority are associated with oncogenesis, DNA damage response, and chromosomal instability [51].

The down-regulated genes set in HSV-tk mice tumor versus that in WT samples, and also in the tumor versus steatotic hepatitis samples, was enriched with lipid metabolic pathways (Figure 4B). Steroid metabolic process, lipid metabolism, and fatty acid metabolism-related gene down-regulation are correlated with dramatic accumulation of neutral lipids and fat in HSV-tk mice liver and tumor tissues. Other work has employed the Clustered Regularly Interspaced Short Palindromic Repeats (CRISPR) system to target the tumor suppressor gene *Pten*, which is a negative regulator of the PI3-Kinase/Akt pathway. Liver-specific knockout of *Pten* in mice induces lipid accumulation and late-onset liver cancer [52,53]. The linkage between the expression of HSV-tk and lipid metabolism disorders in HSV-tk mice is an unanswered question which merits further investigation.

To investigate how the changes learned are related to other transgene mice models, the gene expression signature in HSV-tk mice with HCC was compared to already published data on Notch mice with HCC. Our results show that in the HSV-tk mice, immune-related signals are up-regulated compared to those in *Notch* mice, while genes involved in cell cycle and DNA repair pathways were down-regulated. This suggests that increased expression of immune-inflammation related genes is a characteristic for HSV-tk mice and that inflammation-induced cell cycle activation might be a precursory step to cancer.

There were other studies on the influences of inflammation on liver cancer development in transgenic mice [54]. The inflammatory responses were induced by two carcinogens, 3, 5-diethoxycarbonyl-1, 4-dihydrocollidine (DDC), a non-hereditary hepatotoxin that induces inflammation and fibrosis, and CCl_4_ which causes hepatic lobule centered damage that accompanied by inflammation and fibrosis. The inflammation resulted was induced by chemical toxicity mechanism and would result in strong heterogeneity. On the other hand, what we described here is an investigational platform using HSV-tk mice, which could respond to more biological and physiological stimuli, and are expected to be a good model in the study of the impact of immune-inflammation on the occurrence of liver cancer.

## 5. Conclusions

In summary, our results suggest that the characteristics of HSV-tk mouse with HCC are associated with gene expression related to immune-inflammation and cell cycle. The HSV-tk mouse provides a suitable model for the study of the relationship between immune-inflammation and HCC, and their underlying mechanism for the development of therapeutic application in the future.

## Figures and Tables

**Table 1:** Sequences for primers used by PCR and iPCR.

	Primer Sequence (5′ to 3′)	
	Forward	Reverse
PCR for *HSV-tk*	GTATACCGGTATGCCCACGCTACTGCGG	GATGGCGGTGAAGATGAGG
first-round iPCR	GAGCATGAGGTGACACTACT	CTAGGCTGTGAGGATACA AG
second-round iPCR	ATACCATCATTCCGGACGTG	CTAGGCTGTGAGGATACAAG

Note: iPCR refers to inverse PCR.

**Table 2:** The integration sites of three lines of HSV-tk mice.

Lines of HSV-tk Mice	Chromosomal Location	Intergenic Region	Intron of Gene
TK5	5E1-E2	No	*Rufy3*
TK11	11A1-A2	No	*Abca13*
TK4	8C3-C4	Yes	No

**Table 3:** The incidence of Hepatocellular Carcinoma (HCC) in three lines *HSV-tk* mice.

HSV-tk Lines	Incidence of Tumor Development (%)				
	3 Months	6 Months	9 Months	12 Months	>13 Months
TK5	0 (0/7)	100 (4/4)	100 (10/10)	100 (11/11)	100 (2/2)
TK11	0 (0/4)	0 (0/3)	50 (3/6)	100 (6/6)	100 (6/6)
TK4	0 (0/5)	0 (0/4)	25 (1/4)	40 (6/15)	46 (16/35)

## Supplementary Materials

The following are available online at http://www.mdpi.com/2073-4425/9/8/380/s1, Figure S1: Immunohistochemistry analysis of different types of immune cell, Figure S2: Expression profiles of cell cycle related genes in TCGA HCC/LIHC patients, Figure S3: Expression profiles of Rufy3 and Pak1 in WT, hepatitis and tumor groups, Table S1: Overall health condition of mice included in transcriptome study.
