# Supplementary material for: A Herpes Simplex Virus Thymidine Kinase-Induced Mouse Model of Hepatocellular Carcinoma Associated with Up-Regulated Immune-Inflammatory-Related Signals
Source: Genes (Basel). 2018 Jul 27;9(8):380. doi: 10.3390/genes9080380 (PMC6115908; doi:10.3390/genes9080380)
Supplement: Supplementary file 1 [file genes-09-00380-s001.zip › GongEtAl-SupplmentaryFig&Table-F.docx]

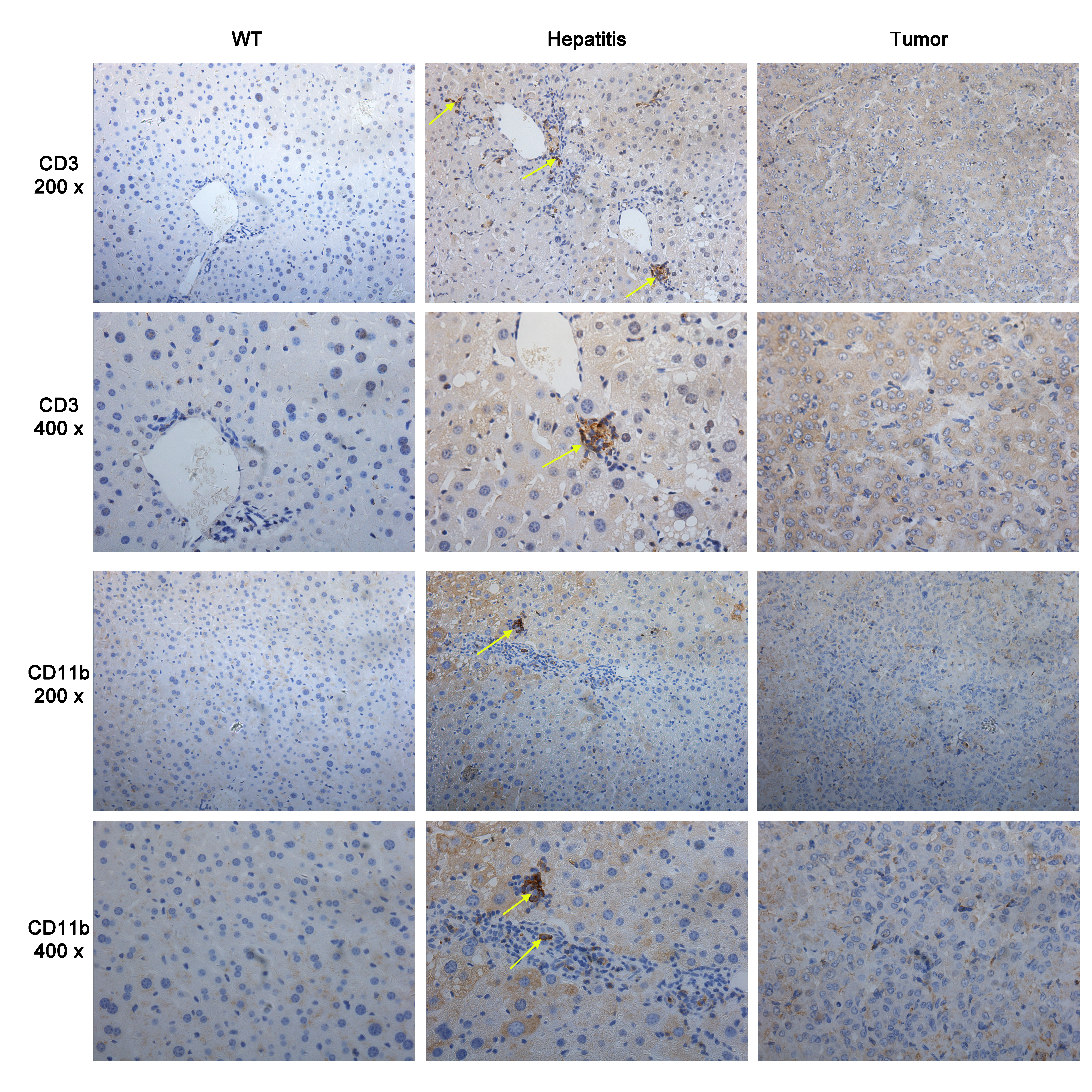


**Figure S1. Immunohistochemistry analysis of different types of immune cell.**

Upper two row images: tissue sections from WT, Hepatitis and Tumor analyzed for CD3^+^ T cell. T cell appeared to be presented only in the Hepatitis group (yellow arrow), but not found in and control (WT) and the Tumor groups (Magnification, x 200 and x400).

Lower two row images: tissue sections from WT, Hepatitis and Tumor analyzed for CD11b^+^ granulocyte. Granulocyte appeared to be present in the Hepatitis group (yellow arrow), but not found in the control (WT) and Tumor groups (Magnification, x 200 and x400).


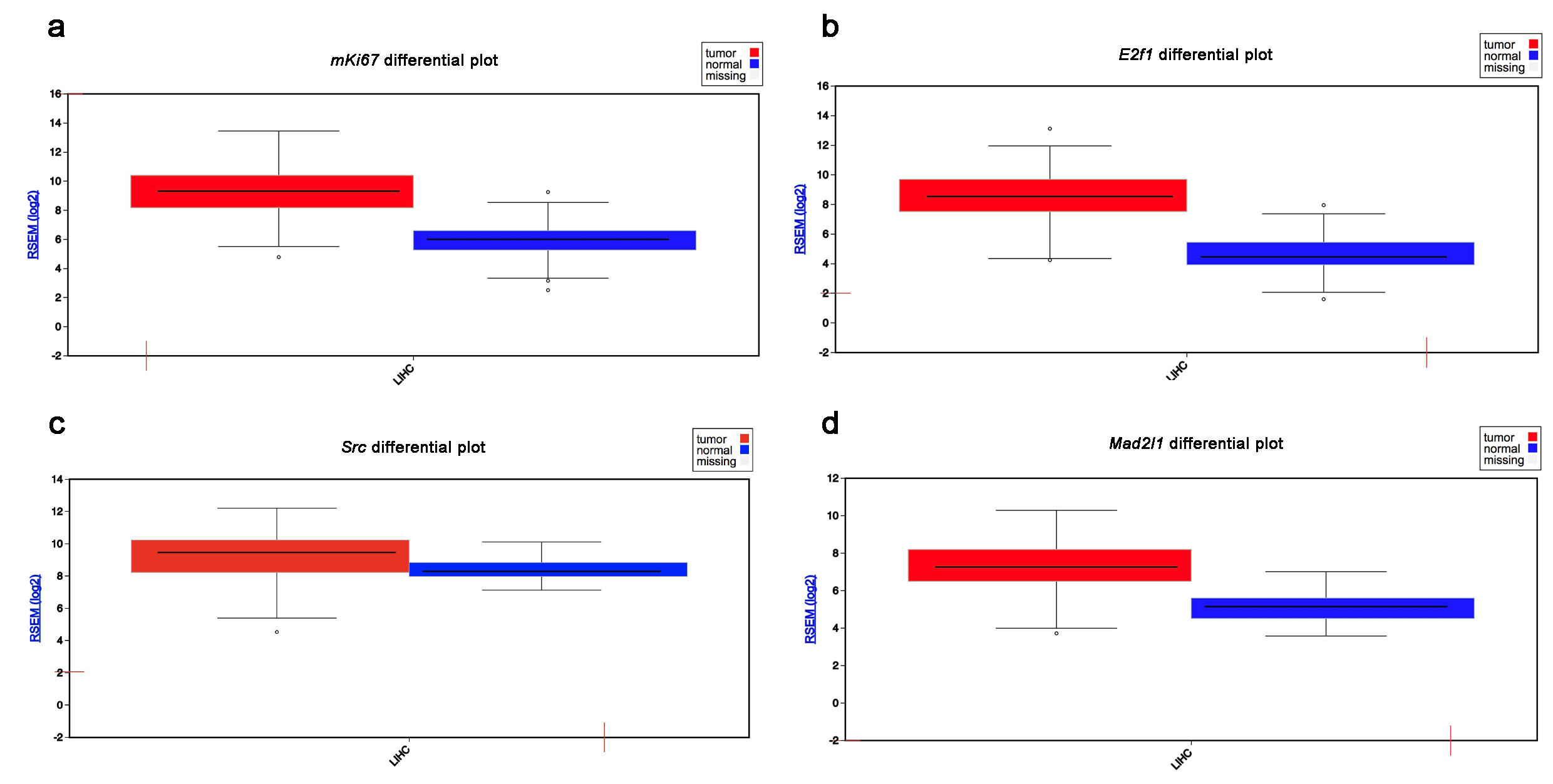


**Figure S2. Expression profiles of cell cycle related genes in TCGA HCC/LIHC patients.**

Red and blue boxes represent tumor and normal samples respectively. Y axis is the log2 RSEM values. Most genes are up-regulated in tumors. a)-d) represent the boxplots for *mKi67*, *E2f1*, *Src* and *Mad2l1*, respectively.


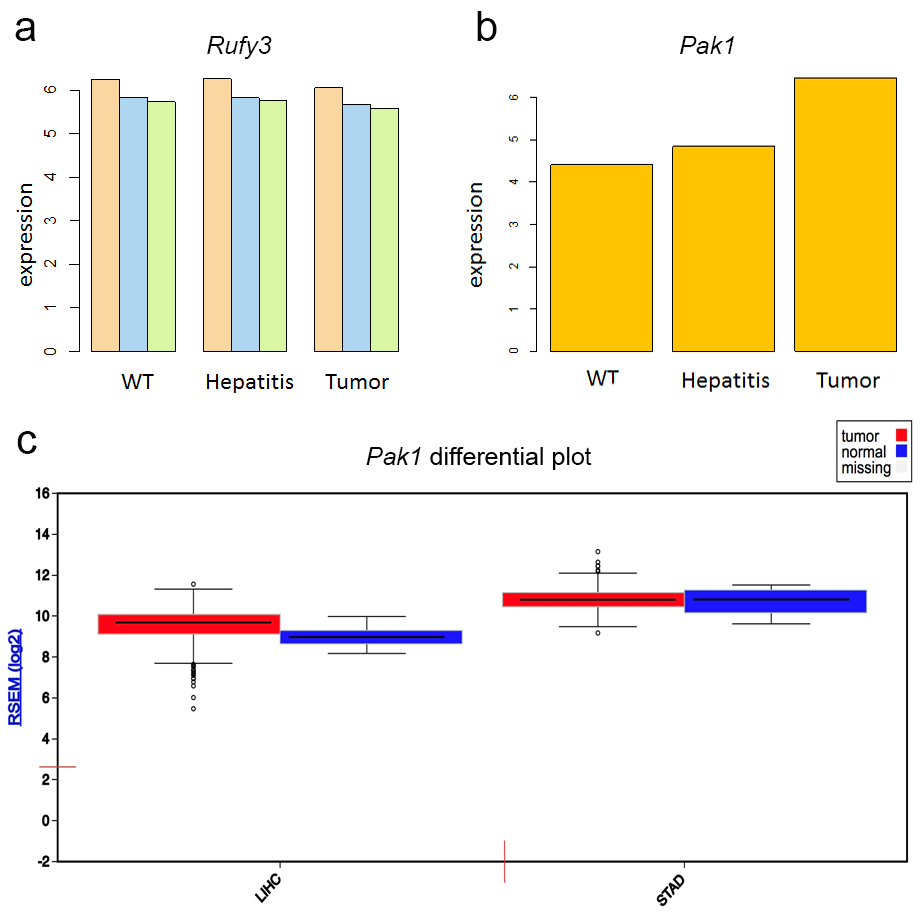


**Figure S3. Expression profiles of *Rufy3* and *Pak1* in WT, hepatitis and tumor groups.**

a) Expression profiles of *Rufy3* in WT, Hepatitis and Tumor. Bars with different color represents different isoforms of *Rufy3* (Brown: NM_001289775, Blue: NM_001289776; Green: NM_027530).

b) Expression profiles of *Pak1* in WT, Hepatitis and Tumor groups.

c) Expression profiles of *Pak1* from the TCGA datasets: LIHC and STAD (liver and gastric cancer). *Pak1* is up-regulated in Liver cancer patients compared to normal samples.

**Table S1. Overall health condition of mice included in transcriptome study**

| Different group | Number of mice | Gender | Overall health condition | Serologic test | |
| --- | --- | --- | --- | --- | --- |
|  |  |  |  | ALT (U/L) | AST (U/L) |
| WT | N081 | F | health | 38 | 116 |
|  | N088 | F |  | 54 | 112 |
|  | N086 | M |  | 40 | 155 |
|  | N149 | M |  | 44 | 131 |
| Hepatitis | TK4-87 | F | A little obesity | 98 | 158 |
|  | TK4-89 | F |  | 124 | 246 |
|  | TK4-90 | M |  | 144 | 132 |
|  | TK4-173 | M |  | 70 | 136 |
| HCC | TK5-192 | F | The liver with tumor in the abdomen is prominent，health condition is not good | 257 | 280 |
|  | TK11-359 | F |  | 601 | 568 |
|  | TK11-100 | M |  | 309 | 367 |
|  | TK11-113 | M |  | 366 | 431 |

F: female M: male
