# Supplementary figures and images for: A Herpes Simplex Virus Thymidine Kinase-Induced Mouse Model of Hepatocellular Carcinoma Associated with Up-Regulated Immune-Inflammatory-Related Signals
Source: Genes (Basel). 2018 Jul 27;9(8):380. doi: 10.3390/genes9080380 (PMC6115908; doi:10.3390/genes9080380)

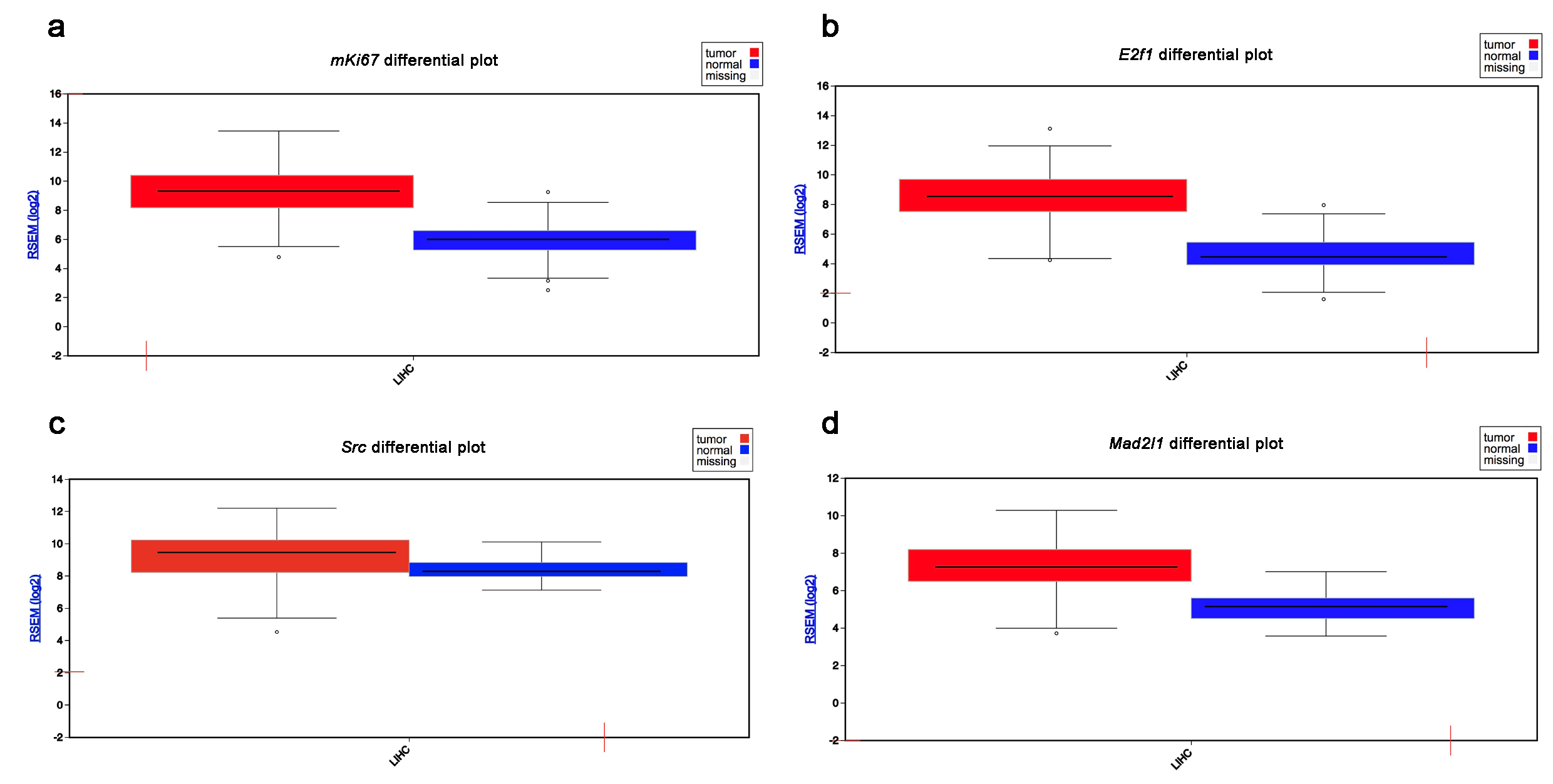

Supplement: Supplementary file 1 [file genes-09-00380-s001.zip › Figure S2-F1.tif]

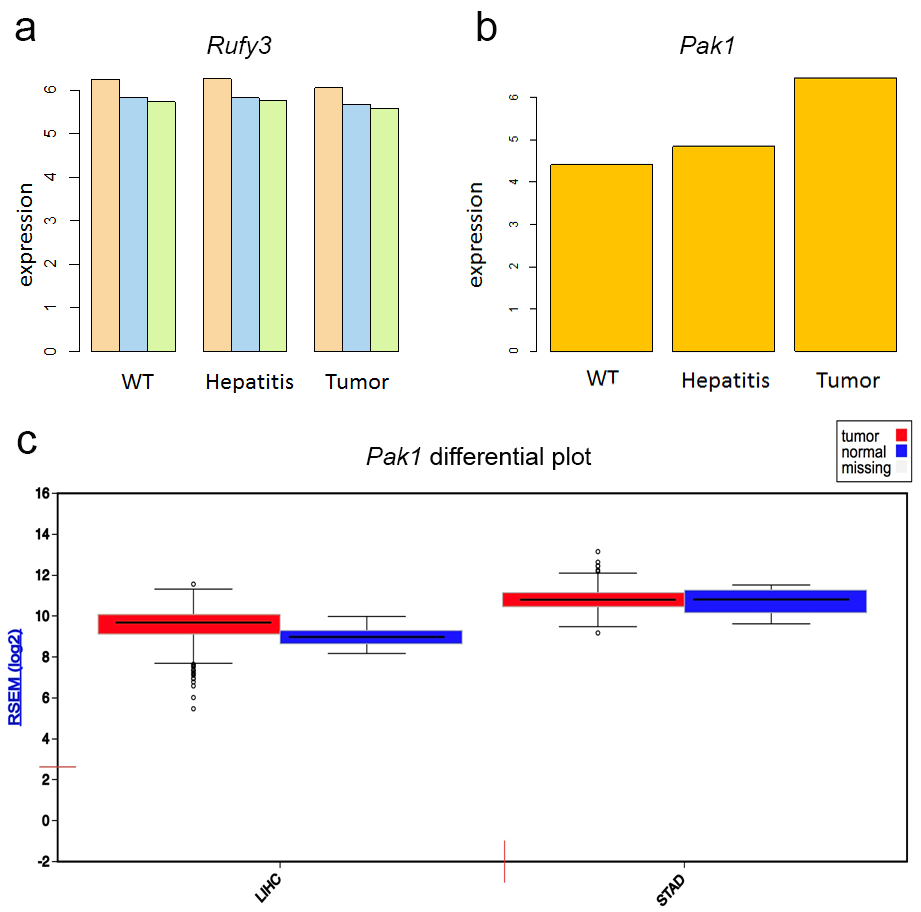

Supplement: Supplementary file 1 [file genes-09-00380-s001.zip › Figure S3-F.tif]
